# Ten quick tips for using the NIH Comparative Genomics Resource

**DOI:** 10.1371/journal.pcbi.1013919

**Published:** 2026-02-11

**Authors:** Eric S. Tvedte, Cecilia Arighi, Matthew B. Carson, Luke V. Rasmussen, Kristi Holmes, Terence D. Murphy

**Affiliations:** 1 National Center for Biotechnology Information, National Library of Medicine, National Institutes of Health, Bethesda, Maryland, United States of America; 2 Department of Computer and Information Sciences, University of Delaware, Newark, Delaware, United States of America; 3 Galter Health Sciences Library & Learning Center, Northwestern University Feinberg School of Medicine, Chicago, Illinois, United States of America; 4 Department of Preventive Medicine, Northwestern University Feinberg School of Medicine, Chicago, Illinois, United States of America; SIB: Swiss Institute of Bioinformatics, SWITZERLAND

## Introduction

The growth of publicly available eukaryotic genomic data has revolutionized both life sciences and biomedical research. By sequencing entire genomes, scientists have accelerated their ability to investigate complex biological systems with greater depth and precision. Genomic data plays a pivotal role in discovering disease-causing mutations, identifying biomarkers for early diagnosis, and uncovering new targets for drug development. Moreover, integrating genomic data with other -omics technologies, such as transcriptomics and proteomics, offers a comprehensive view of cellular functions, paving the way for personalized medicine.

The NIH Comparative Genomics Resource (CGR; https://www.ncbi.nlm.nih.gov/cgr/) [1] led by the National Center for Biotechnology Information (NCBI) is a groundbreaking initiative aimed at enabling researchers to explore and compare genomic data across diverse eukaryotic species. This resource includes genomic data, tools, and community resources to support comparative genomics studies, helping scientists better understand evolutionary relationships and accelerating biomedical research discovery. Its user-friendly platform ensures that data from a wide range of organisms is accessible to researchers worldwide in both human- and machine-readable formats.

**Fig 1:**
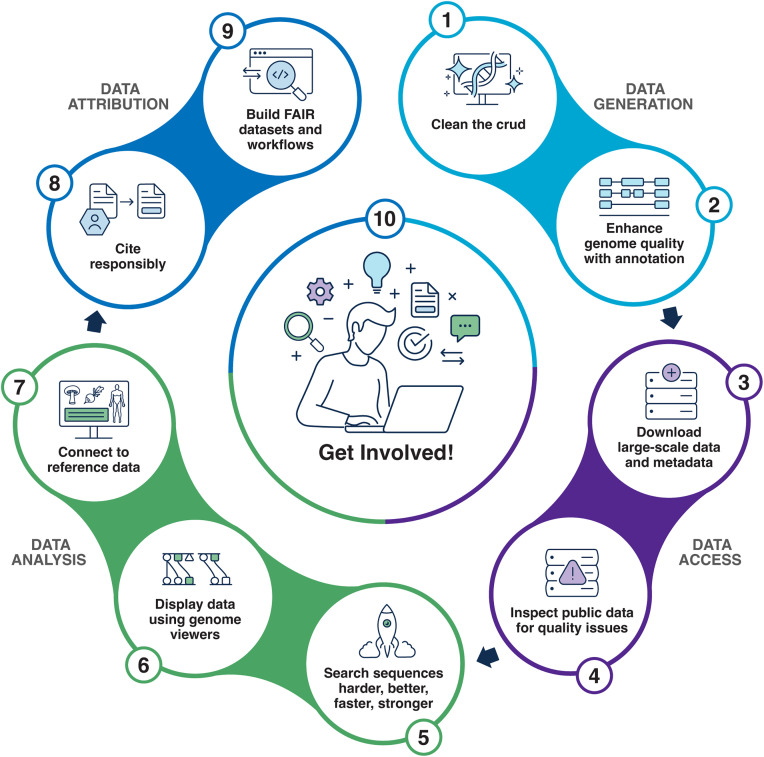
The Ten Quick Tips for conducting genomics research using the NIH Comparative Genomics Resource can utilize NCBI data and tools throughout the lifecycle of a research project.

The recent “Ten Simple Rules for using public biological data in your research” [2] provides an excellent discussion of the basic principles of using public data. Here, we discuss Ten Quick Tips for utilizing CGR data and tools in genomics research as a guide for both new and experienced users of NCBI resources. CGR resources can be beneficial throughout a project’s lifecycle starting with user-generated data (Fig 1). Prioritization of the steps (tips) is up to the researcher.

## Tip 1: Clean up the crud

When assembling new genomes, post-assembly curation is typically needed to fix errors. Genome assemblies often contain contaminants (a.k.a. ‘crud’) from non-target organisms and/or sequencing adaptors. You can identify and remove contaminants using the NCBI Foreign Contamination Screen (FCS; https://github.com/ncbi/fcs) [3]. The crud itself may be a valuable source of genome data for cobiont organisms that can be assembled to understand potential host-cobiont relationships [4]. And don’t forget to document your curation steps and quality metrics to help future researchers assess the quality of your genome assembly [5–7].

## Tip 2: Enhance genome quality with annotation

To identify functional elements in the genome sequence, annotation pipelines use ab initio or evidence-based methods or an integration of the two, accounting for repeat content [8]. The NCBI Eukaryotic Genome Annotation Pipeline (EGAP) (https://www.ncbi.nlm.nih.gov/refseq/annotation_euk/process/) has been used for 25 years to generate genome annotations for RefSeq genomes. To support community annotation efforts, you can now use EGAPx (https://github.com/ncbi/egapx), a standalone adaptation of EGAP for annotating genomes that can be readily submitted to GenBank. Whether you use EGAPx or another annotation program, you should submit your genome and annotation to an archival database that is part of the International Nucleotide Sequence Database Consortium (INSDC) [9]; submission promotes data sharing and reuse, and helps ensure your efforts make an impact for years to come.

## Tip 3: Download large-scale data and metadata

NCBI GenBank is growing continuously, containing 34 trillion base pairs from over 4.7 billion nucleotide sequences for 581,000 formally described species as of 2025 [10]. Large datasets can increase the confidence in observed patterns. More genomes at different evolutionary distances can be valuable in identifying and confirming the evolutionary context of an observation. NCBI Datasets [11] provides scalable access to gene, genome, and taxonomy information through multiple interfaces (web, command-line, API). A common use case in comparative genomics is to download multiple genomes from a taxonomic group of interest, including one or more reference genomes (see Tip 7) that NCBI has identified as the “best” for a species (https://www.ncbi.nlm.nih.gov/datasets/docs/v2/glossary/). You can use data retrieved from NCBI Datasets in a variety of contexts, such as building catalogs of sequences of a defined area of interest [12], investigating gene variation to understand evolutionary patterns [13], or as a case study for a new bioinformatic tool [14]. All data retrieved through NCBI Datasets includes a comprehensive metadata report. These reports follow documented schemas that provide standardized, structured descriptions of biological data stored at NCBI. Consider the biological questions of interest when choosing the taxonomic scope of the dataset: restricting to closely related taxa enables detection of recent evolutionary events due to high sequence similarity, while including more distantly related taxa allows investigation of deeper evolutionary timescales and can highlight conserved sequences over long periods of time.

## Tip 4: Inspect public data for quality issues

Although the growth in public genomic data is mostly beneficial, there are challenges in working with data with inconsistent features and/or quality. In extreme situations, using incorrect data can actively hinder your biological interpretations of results, so don’t assume all publicly-available data is correct. You can use public NCBI FCS reports updated daily (https://ftp.ncbi.nlm.nih.gov/genomes/ASSEMBLY_REPORTS/) to select genomes of interest based on your acceptable contamination level threshold or remove sequences assigned as contaminants. The scalable data access of NCBI Datasets can potentially expose systematic errors such as gene family mis-annotations [15]. Filters in NCBI Datasets queries can improve your data quality consistency (e.g., retain chromosome-level genome assemblies, retain annotated genomes, remove atypical/contaminated genomes).

Following data download, you should perform additional quality checks, such as examining the metadata to ensure the data is appropriate for your use case as well as verifying the integrity of downloads via checksums or other means (see Rules 2 and 7 from [2] for additional details). Commonly used quality metrics such as contig N50 and BUSCO [16] scores can also be used to remove low-quality outliers.

## Tip 5: Search sequences harder, better, faster, stronger

The default BLAST [17,18] parameters are tailored for a balance between sensitivity and speed, but you might consider adjusting parameters for your specific use case. Examples include the choice of BLAST algorithm (e.g., megablast, discontinuous megablast, and blastn in nucleotide BLAST searches), word size, and filtering on low-complexity regions. It is important to understand the chosen parameters in order to effectively interpret the results; parameters such as max_target_seqs and evalue are commonly misinterpreted [19–21].

New BLAST databases have been developed as part of the CGR project. Understanding how BLAST database size and content can influence search outcomes is crucial for tailoring performance [22]. While the use of larger databases may produce more comprehensive results, sequence redundancy can increase the database size significantly slowing run times without any meaningful change in the results. The BLAST nucleotide database (nt) is currently 2.5 trillion letters and has a doubling rate of less than a year. Conversely, default searches now use the core nucleotide database (core_nt) that is less than half the size of the standard nt database, enabling faster searches with minimal impact on top-hit quality [23]. Default searches of protein databases use the new ClusteredNR database [24], which reduces the redundancy of protein sequences in the standard protein database (nr) into clusters with 90% identity/ 90% length thresholds. Searches using ClusteredNR are faster and provide results across a wider range of organisms and evolutionary distances. Additional options for customizing database content include by source (e.g., RefSeq, WGS, Swiss-Prot), type (rRNA/ITS databases) and organism(s) using tax-ids. Your research goals should help define your search strategies, and by experimenting with BLAST you will be able to design searches to achieve the appropriate balance of match distance, sensitivity, and speed.

Web BLAST searches use a fixed amount of CPU time. If your searches consistently exceed the CPU limit, you can download BLAST databases from NCBI and use BLAST+ executables (https://ftp.ncbi.nlm.nih.gov/blast/executables/blast+/LATEST/) or try ElasticBLAST [25] on the cloud.

## Tip 6: Display data using genome viewers

Genome data visualizations can uncover and communicate complex patterns effectively. The NCBI Genome Data Viewer (GDV) [26] is a linear genome browser that can display gene annotation, experimental data (e.g., RNA-seq), and alignments to other genomes to understand how genome sequence correlates to function. Try viewing pairwise whole genome sequence alignments using the NCBI Comparative Genome Viewer (CGV) [27]. CGV views highlight large-scale structural rearrangements such as duplications, deletions, and inversions, allowing you to assess gene synteny and analyze changes that may have contributed to differences in biology or phenotype. When you’re ready to publish, GDV and CGV both include high-quality SVG download options to help visualize your story.

If you have a gene or genomic region of interest and want to compare across many species, the new NCBI Multiple Comparative Genome Viewer (MCGV; https://www.ncbi.nlm.nih.gov/mcgv/) can display whole-genome multiple alignments created by the research community. MCGV provides alignment and conservation summaries to help you find locations that are well conserved or divergent.

## Tip 7: Connect to reference data

A focal point of the CGR project is the support for high-quality reference sequences for comparative genomics analyses. The NCBI RefSeq resource (https://www.ncbi.nlm.nih.gov/refseq/) is a stable, non-redundant collection of reference sequences for genomes, transcriptomes, and proteins [28–30]. The RefSeq collection is generated from a combination of automatic processes, manual curation, and community collaboration. As of November 2025 there are over 2200 eukaryote species with whole genomes represented in RefSeq. By using well-established references, you can perform analyses that are understandable and reproducible by the scientific community. Transcriptomics, proteomics, sequence variation, and multiple species alignments can all be informed by annotated references, enabling new biomedical discoveries. You can also use RefSeq sequences as connection points to many other NCBI resources. Try using RefSeq accessions to find gene-centric information on the Gene database [31], search against databases of RefSeq sequences using BLAST, collect orthologs using NCBI Datasets, and visualize annotated features using GDV and CGV.

## Tip 8: Cite responsibly

By giving credit to researchers whose data you use in comparative genomics analysis, you promote the sharing of data in open databases [2,32]. Check how the original authors who produced the data and/or code want to be credited [32]. If there is no specific guidance, you should reference a persistent data object identifier (DOI) corresponding to the stored data and/or the original publication where the data was produced. NCBI Genome Datasets pages include links to BioProject metadata as well as links to relevant publications that may contain relevant citations. There is also information on how to cite NCBI services and databases [33,34]. Publications that share and access data in public databases tend to be more highly cited than those that do not [2,35,36], so taking the time to give appropriate credit benefits you as well as the original producer of the data.

## Tip 9: Build FAIR datasets and workflows

The FAIR (Findable, Accessible, Interoperable, Reusable) Principles are widely accepted best practices for sharing research output [37,38]. FAIR data enhances discovery, access, and integration with other data by providing an identifiable location, proper context, and guidance for reuse to other researchers. To support FAIR principles using CGR tools and data:

- **Findability:** Use persistent identifiers with rich metadata to improve the findability for newly generated data (Tips 1 and 2) and public data (Tip 3). NCBI assigns permanent identifiers (accession numbers) for all deposited genomic data including BioProjects, assemblies, and annotated proteins which can be used as search strings on NCBI databases. When your workflows use CGR tools, you should provide relevant software versions to enhance the findability of documentation for the original software.
- **Accessibility:** Submitting data and metadata to NCBI enables open and free access using standardized procedures (web, command-line, API). If data types are not supported by NCBI, your data should be downloadable from a general-purpose repository and include documentation for acquiring and incorporating your data into research workflows. Research software that uses CGR tools and data should also be open source.
- **Interoperability:** By preparing data in standard formats (e.g., FASTA/FASTQ, GFF, MAF) alongside rich metadata in JSONL and TSV, you can access and compare datasets from a variety of sources. By reading, writing, and exchanging data using community standard formats, research software can interact with other research software, for example through NCBI Datasets APIs.
- **Reusability:** Enriching your data and software with metadata including relevant attributes appropriate for your data types, licensing information, and references to other data and/or software will support reusability. Reusability can be enhanced using containers (e.g., Docker, Singularity). For example, the NCBI FCS (Tip 1) has been incorporated into multiple genome assembly evaluation workflows [39–41].

It is important to note that reusability does not necessarily guarantee reproducibility. For example, CGR tools can be FAIR compliant but data access (e.g., NCBI Datasets) and data analysis (e.g., BLAST) may return different results over time following updates to data resources. To support reusability and transparency, researchers should report:

- Dataset author, publication year, name, and version used (or date accessed if no version)
- Software author, name, and version used (and citation if available)
- The query itself (including query string if applicable, parameters, or options), execution date of the query, and the context (what was the research question that was answered?)
- A permanent identifier or direct access link to the results if available

## Tip 10: Get involved!

Providing feedback is a worthwhile effort; CGR continues to benefit from community input to enhance its tools. Researchers can submit feedback, participate in surveys, or join discussions through forums, workshops, and user meetings. The easiest way to provide feedback is through the yellow “Feedback” button in the lower right-hand corner of CGR and other NCBI webpages.

A wide range of curricula, tutorials, and workshops are freely available (https://www.ncbi.nlm.nih.gov/cgr/) to support use of CGR resources, as are links to published papers which have applied these resources. Individuals can subscribe to the NCBI Insights newsletter (https://ncbiinsights.ncbi.nlm.nih.gov) for CGR-specific content and receive updates on new CGR tools, datasets, and research findings. Email CGR at cgr@nlm.nih.gov to teach a workshop, partner on a webinar, or discuss other ideas you may have to foster information sharing and feedback. By taking advantage of these opportunities, researchers can both contribute to and stay informed about the latest developments in comparative genomics.

## Conclusions

The NIH CGR plays a pivotal role in translating comparative genomics into actionable insights for basic science and applied research. By uncovering shared genetic pathways and evolutionary patterns, CGR supports the identification of genes and genetic variations associated with specific traits or diseases. Such insights have profound implications for understanding human health, agriculture, and environmental biology. The integration of community data with NCBI tools enhances the utility of the resource, empowering researchers to generate robust hypotheses and validate findings. As a centralized hub for comparative genomics, CGR fosters collaboration and accelerates discoveries, making it an asset in the pursuit of scientific innovation and the development of solutions to complex biological and medical challenges.
